# Supplementary material for: The Range‐Resident Logistic Model: A New Framework to Formalise the Population‐Dynamics Consequences of Range Residency
Source: Ecol Lett. 2025 Dec 11;28(12):e70269. doi: 10.1111/ele.70269 (PMC12698032; doi:10.1111/ele.70269)
Supplement: Supplementary file 1 — Data S1: ele70269‐sup‐0001‐Supinfo.pdf. [file ELE-28-0-s001.pdf]

Supporting Information

A The crowding index for home range centers.

In this appendix, we derive the expression for the crowding index based on home-range centers, Eq. (8) of the main text, starting from its definition in terms of the instantaneous organism location, Eq. (5) of the main text.

The position of a focal organism, i, and the displacement between this organism i and a neighbor j, both at time t, can be represented by two random variables $X_t^{(i)}$ and $U_t^{(i,j)} = X_t^{(j)} - X_t^{(i)}$, respectively. Each of these random variables has a probability density function (PDF), $f_{X_t^{(i)}}(\boldsymbol{x})$ and $f_{U_t^{(i,j)}}(\boldsymbol{u})$, respectively. Similarly, we denote each individual's home-range center as $\bar{X}^{(i)}$, the displacement of the organism from its home range center at time t as $Y_t^{(i)}$, and the displacement between a pair of organisms' home-range centers as $\bar{U}^{(i,j)}$. Using these definitions, the displacement between organisms is

$$U_t^{(i,j)} = X_t^{(j)} - X_t^{(i)} = X_t^{(j)} - X_t^{(i)} \pm \overline{X}^{(i)} \pm \overline{X}^{(j)} = Y_t^{(j)} - Y_t^{(i)} + \overline{U}^{(i,j)} . \tag{A.1}$$

Because the PDF of a sum of random variables is the convolution of the PDFs of the random variables, the PDF of the displacement between organisms at time t is given by the convolution of PDF associated to $Y_t^{(j)}$, $-Y_t^{(i)}$ and $\overline{U}^{(i,j)}$. Mathematically, and using f to denote all PDFs, we can write this relationship as

$$f_{U_t^{(i,j)}}(\boldsymbol{u}, t) = \left(f_{-Y_t^{(i)}} * f_{Y_t^{(j)}} * f_{\overline{U}^{(i,j)}}\right)(\boldsymbol{u}, t). \tag{A.2}$$

Additionally, the distribution of displacements between organisms i and j, averaged over all possible pairs of organisms, is directly related to the pair correlation function $g_2(\boldsymbol{u}, t)$

$$g_2(\boldsymbol{u}, t) = \frac{A}{N(N-1)} \sum_{i \neq j} f_{U^{(i,j)}}(\boldsymbol{u}, t) \tag{A.3}$$

$$= \frac{A}{N(N-1)} \sum_{i \neq j} \left(f_{-Y_t^{(i)}} * f_{Y_t^{(j)}} * f_{\overline{U}^{(i,j)}}\right)(\boldsymbol{u}, t) . \tag{A.4}$$

For range-resident organisms, the distribution of their position around its center tends to a constant asymptotic distribution directly related to its movement behavior, i.e. $f_{Y^{(i)}_{t\to\infty}} = f_{Y^{(i)}}$ for any organism i. If we further assume that the home ranges of organisms are identical ($f_{Y^{(i)}} = f_{Y^{(j)}} = f_Y \, \forall \, i, j$) and symmetric ($f_{-Y} = f_Y$), we can use the linearity of the convolution to write

$$g_2(\boldsymbol{u}, t) = \left(f_Y * f_Y * \frac{A}{N(N-1)} \sum_{i \neq j} f_{\overline{U}^{(i,j)}} \right)(\boldsymbol{u}, t) \tag{A.5}$$

$$g_2(\boldsymbol{u}, t) = \left(f_Y * f_Y * g_2^{\mathrm{HR}}(\boldsymbol{u}, t)\right)(\boldsymbol{u}, t) , \tag{A.6}$$

where we can identify $g_2^{\mathrm{HR}}(\boldsymbol{u}, t) = \frac{A}{N(N-1)} \sum_{i \neq j} f_{\overline{U}^{(i,j)}}$ with the home-range center pair correlation function.

Finally, using Eq. (A.6), we can rewrite the crowding index as

$$c(t) = \int_\Omega \mathcal{K}^c(\boldsymbol{u}) g_2(\boldsymbol{u}, t) \mathrm{d}\boldsymbol{u} \tag{A.7}$$

$$= \int_\Omega \mathcal{K}^c(\boldsymbol{u}) \left(f_Y * f_Y * g_2^{\mathrm{HR}}(\boldsymbol{u}, t) \right) (\boldsymbol{u}, t) \mathrm{d}\boldsymbol{u} \tag{A.8}$$

$$= \int_\Omega \left(\mathcal{K}^c * f_Y * f_Y \right) (\boldsymbol{u}) g_2^{\mathrm{HR}}(\boldsymbol{u}, t) \mathrm{d}\boldsymbol{u} \tag{A.9}$$

$$c_{\mathrm{HR}}(t) = \int_\Omega \mathcal{K}^{\mathrm{HR}}(\boldsymbol{u}) g_2^{\mathrm{HR}}(\boldsymbol{u}, t) \mathrm{d}\boldsymbol{u}\,, \tag{A.10}$$

where we define the home-range competition kernel $\mathcal{K}^{\mathrm{HR}}(\boldsymbol{u}) = \left(\mathcal{K}^c * f_Y * f_Y \right) (\boldsymbol{u})$ and we denote the crowding index with the subscript HR to highlight that it is calculated using the distribution of HR centers instead of the using the spatial distribution of organisms.

B Measurement of the crowding index using spatial information.

This appendix explains how to measure the crowding index from simulation data. A simulation snapshot showing the spatial distribution of organisms can be described as a N-point pattern $P = \{\boldsymbol{x}_i\}$ in which $\boldsymbol{x}_i$ denotes the coordinates of the i-th point. Ignoring edge corrections because we are working with toroidal spatial domains, the pair correlation function of such point pattern is (Baddeley *et al.* 2016; Wiegand & Moloney 2014):

$$\hat{g}_2(\boldsymbol{u}) = \frac{1}{N(N-1)} \sum_{i \neq j} \delta \left(\boldsymbol{u} - \boldsymbol{x}_j + \boldsymbol{x}_i \right) \,, \tag{B.1}$$

where $\delta(\boldsymbol{u})$ is the Dirac delta distribution and the summation is over all possible pairs of organisms. Substituting equation (B.1) into Eq. (8) of the main text we get

$$\hat{c} = \frac{1}{N(N-1)} \sum_{i \neq j} \int_\Omega \mathcal{K}^c(\boldsymbol{u}) \delta \left(\boldsymbol{u} - \boldsymbol{x}_j + \boldsymbol{x}_i \right) \mathrm{d}\boldsymbol{u} \tag{B.2}$$

$$= \frac{1}{N(N-1)} \sum_{i \neq j} \mathcal{K}^c \left(\boldsymbol{x}_j - \boldsymbol{x}_i \right) \,, \tag{B.3}$$

which can be used to measure the crowding index in the population, given a spatial distribution of organisms and for a known competition kernel.

Similarly, we apply the same procedure to compute the crowding index using the spatial pattern of home-range centers and space utilization functions. We first compute the home-range competition kernel $\mathcal{K}^{\mathrm{HR}}(\boldsymbol{u})$. Since the asymptotic displacement from the home-range center of the organisms following OU movement and the competition kernel $\mathcal{K}^c$ we implemented are Gaussian distributions centered at zero, their convolution is also a Gaussian distribution centered at zero. The crowding index based on home-range centers can then be computed as

$$\hat{c} = \frac{1}{N(N-1)} \sum_{i \neq j} \mathcal{K}^{\mathrm{HR}} \left(\bar{\boldsymbol{x}}_j - \bar{\boldsymbol{x}}_i \right) \,, \tag{B.4}$$

where $\bar{\boldsymbol{x}}_i$ denotes the position of the home range center of the i-th organism.

We note that the derivation above allows for asymmetric, organism-specific, and pair-specific interaction kernels by taking $\mathcal{K}^c \to \mathcal{K}^c_{ij}$. Thus, the proposed crowding index is compatible with asymmetric interactions, as seen in (Fagan *et al.* 2024), where the outcome of an encounter between two coyotes was asymmetric, leading to a one-sided shift in their home range overlap.

C Supplementary Figures

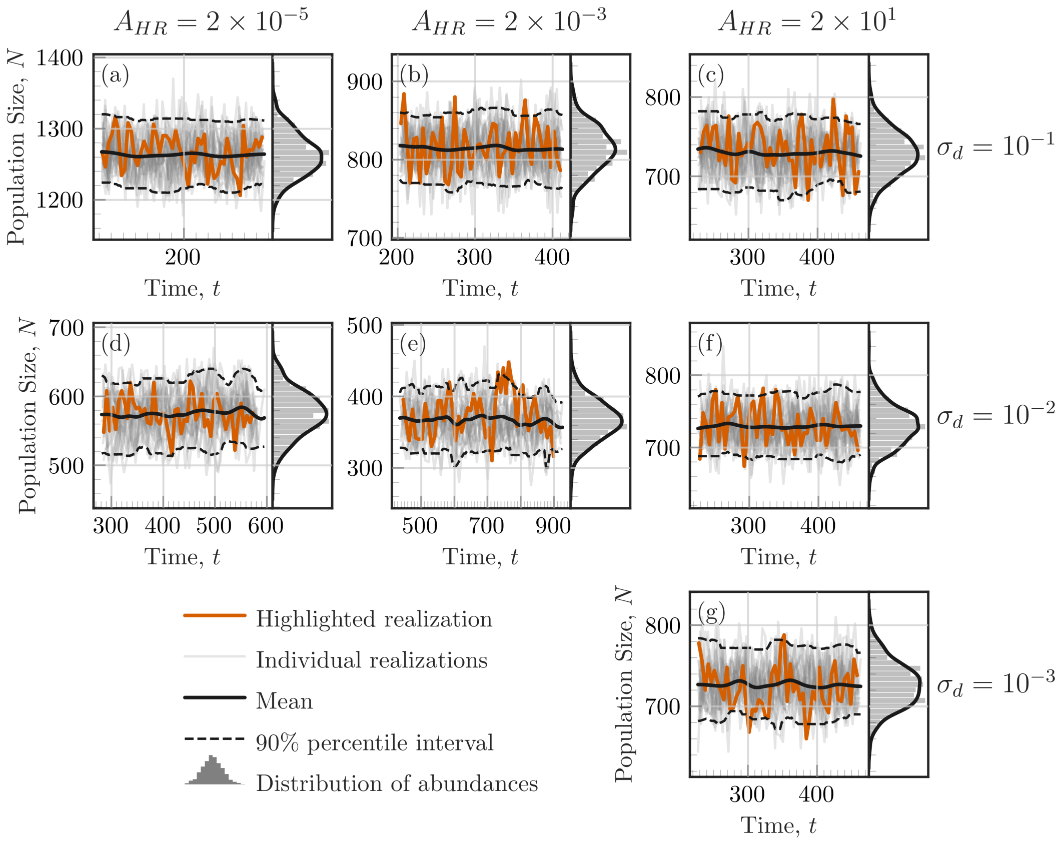

Figure C.1: Diagnostic plots for stationarity of abundance. The abundance (y-axis) recorded in each realization as a function of simulation time (x-axis) is shown as light-gray lines. An arbitrary realization is highlighted in orange for easier visualization. The mean and 90% percentile interval of the abundance over 20 realizations are shown as full and dashed black lines, respectively. The distribution of recorded abundances over the measuring time (time range shown in x-axis of each panel) is shown as a histogram on the right. The data for this figure are excerpted from Fig. 2 on the main text.

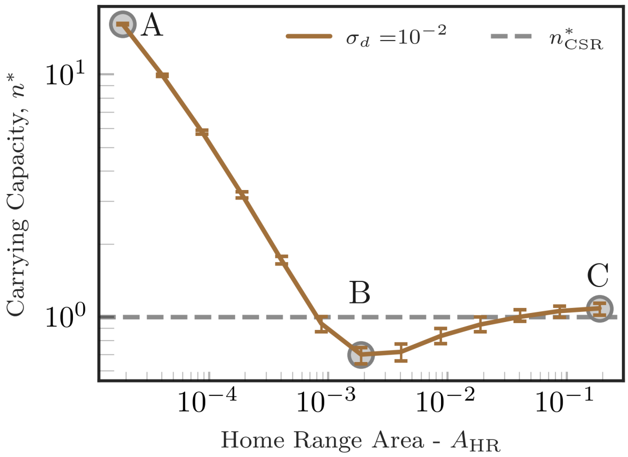

Figure C.2: Home-range size alone can drive steady-state population sizes above or below the homogeneous carrying capacity. Increasing home-range size while all other spatial scales constant (dispersal and competition) can drive the stationary-state population size from much higher than predicted for a completely spatial random population, n^*_{CSR} (A), to smaller (B), to finally match n^*_{CSR} (C). Standard deviations of competition and dispersal kernels were set to $\sigma_q = 10^{-3}$ and $\sigma_d = 10^{-2}$, respectivelly.

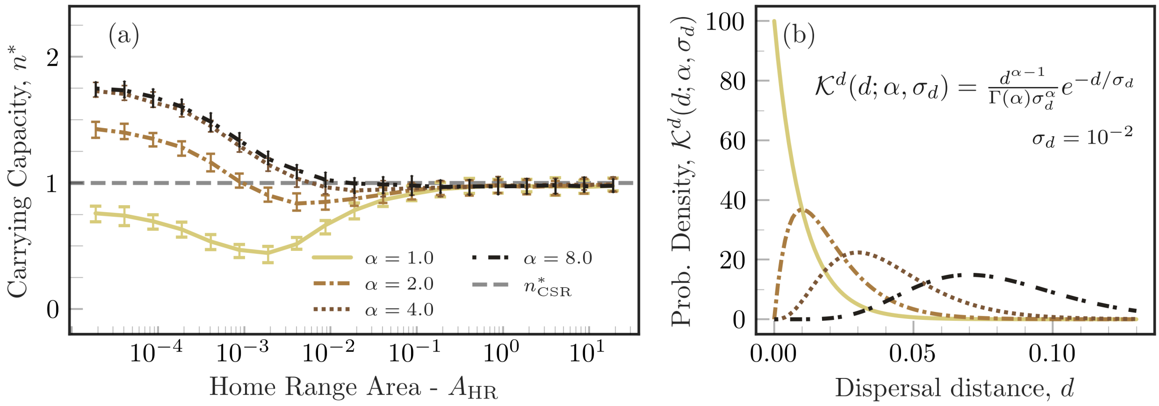

Figure C.3: Carrying capacity versus home-range size for gamma-distributed dispersal distances. The results obtained by varying the shape parameter of the gamma distribution mirror those obtained by varying the standard deviation of the bivariate normal distribution. For this analysis, the dispersal kernel was defined such that the radial distance from the parent's home range center follows a gamma distribution with shape parameter $\alpha \in [1, 2, 4, 8]$ (different colors and line styles as indicated in the legend of panel (a)) and constant dispersal and competition scale parameters $\sigma_d = \sigma_q = 10^{-2}$. The direction of dispersal was assumed to be uniformly distributed in $[0, 2\pi)$. (a) Carrying capacity measured in the simulations and scaled by the carrying capacity of the non-spatial model, as in Fig. 2 of the main text. (b) Dispersal probability density function for the dispersal kernels used in (a). Color lines correspond to values of α as indicated in the legend of panel (a). The dispersal and competition scales remained constant for all simulations, $\sigma_d = \sigma_q = 10^{-2}$.

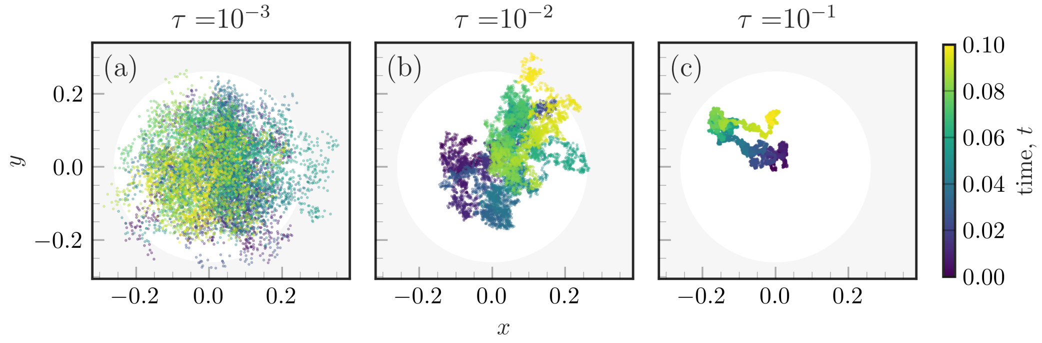

Figure C.4: The effect of home range crossing time on how fast OU-moving individuals cover their entire home range. Organisms take longer to explore their entire home range when the home-range crossing time τ increases (from panel a to c), keeping home-range size fixed, $A_{\mathrm{HR}} = 0.20(a.u.)$. In the three panels, we simulated a single trajectory for a total of $t = 0.1(a.u.)$ with time increments of $dt = 10^{-5}(a.u.)$. For smaller values of τ (panel a), the organism effectively explores the entire HR. However, if τ increases but we keep the simulation time constant, the organism explores a more restricted region of its home range. In the limit where τ tends to zero, the trajectory loses all the autocorrelation between consecutive locations, which becomes an independent and identically distributed variable (IID). Consequently, the organism can be found anywhere within the home range at each time step.

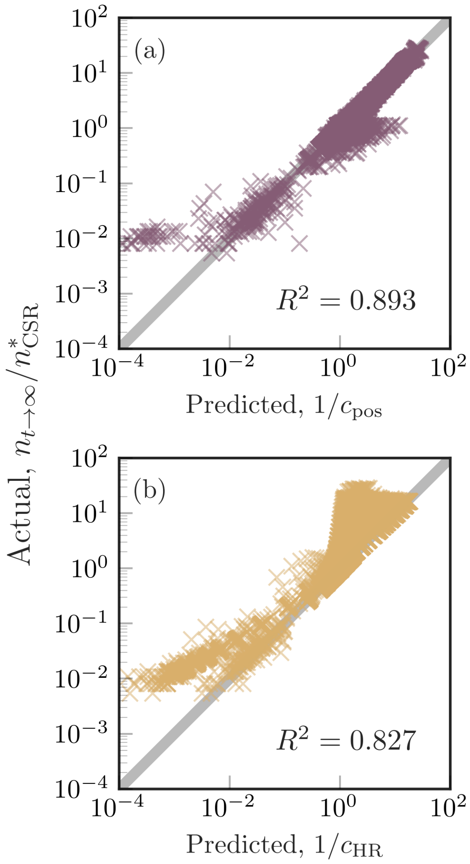

Figure C.5: Accuracy of the crowding index for broader dataset. The crowding index measured using only information about spatial scales across all the simulations matches the measurement of the carrying capacity of the population. The abundance of the population in the simulation normalized by the carrying capacity of a homogeneous population (y-axis) is compared to the inverse of the crowding coefficient (x-axis). (a) The crowding index was measured using the actual position of the organisms as the average local density experienced by each organism in a snapshot of the simulation. (b) The crowding index was measured based on the expected mortality rate an organism experiences given its home-range size and the distances between its home range center and that of its neighbors. The complete agreement (1-1 line) is represented as a gray line in the background of each panel and the R^2 is reported as an indicative of the goodness of fit. No data was filtered out of the analysis, apart from simulations in which extinction was observed.

References

Baddeley, A., Rubak, E. & Turner, R. (2016). *Spatial Point Patterns: Methodology and Applications with R.* Champan & Hall/CRC Interdisciplinary Statistics Series. CRC Press, Taylor & Francis Group, Boca Raton.

Fagan, W.F., Krishnan, A., Liao, Q., Fleming, C.H., Liao, D., Lamb, C. *et al.* (2024). Intraspecific encounters can lead to reduced range overlap. *Movement Ecology*, 12, 1–13.

Wiegand, T. & Moloney, K.A. (2014). *Handbook of Spatial Point-Pattern Analysis in Ecology.* CRC Press, Taylor & Francis Group, Boca Raton.
